# Deciphering the dynamics of methicillin-resistant *Staphylococcus aureus* biofilm formation: from molecular signaling to nanotherapeutic advances

**DOI:** 10.1186/s12964-024-01511-2

**Published:** 2024-03-22

**Authors:** Nirmeen Aboelnaga, Salma W. Elsayed, Nehal Adel Abdelsalam, Salma Salem, Nehal A. Saif, Manar Elsayed, Shehab Ayman, Maha Nasr, Mohamed Elhadidy

**Affiliations:** 1 Center for Genomics, Helmy Institute for Medical Sciences, Zewail City of Science and Technology, Giza, Egypt; 2 Biomedical Sciences Program, University of Science and Technology, Zewail City of Science and Technology, Giza, Egypt; 3 Department of Microbiology & Immunology, Faculty of Pharmacy, Ain Shams University, Cairo, Egypt; 4 Department of Microbiology and Immunology, Faculty of Pharmacy, Cairo University, Cairo, Egypt; 5 Department of Pharmaceutics and Industrial Pharmacy, Faculty of Pharmacy, Ain Shams University, Cairo, Egypt; 6 Department of Bacteriology, Mycology and Immunology, Faculty of Veterinary Medicine, Mansoura University, Mansoura, Egypt

**Keywords:** Methicillin-resistant *Staphylococcus aureus*, Autoinducers, Antibiofilm agents, Biofilm formation, Biofilm-mediated resistance, Quorum sensing, Quorum inhibitors, Nanotherapeutics

## Abstract

Methicillin-resistant *Staphylococcus aureus* (MRSA) represents a global threat, necessitating the development of effective solutions to combat this emerging superbug. In response to selective pressures within healthcare, community, and livestock settings, MRSA has evolved increased biofilm formation as a multifaceted virulence and defensive mechanism, enabling the bacterium to thrive in harsh conditions. This review discusses the molecular mechanisms contributing to biofilm formation across its developmental stages, hence representing a step forward in developing promising strategies for impeding or eradicating biofilms. During staphylococcal biofilm development, cell wall-anchored proteins attach bacterial cells to biotic or abiotic surfaces; extracellular polymeric substances build scaffolds for biofilm formation; the *cidABC* operon controls cell lysis within the biofilm, and proteases facilitate dispersal. Beside the three main sequential stages of biofilm formation (attachment, maturation, and dispersal), this review unveils two unique developmental stages in the biofilm formation process for MRSA; multiplication and exodus. We also highlighted the quorum sensing as a cell-to-cell communication process, allowing distant bacterial cells to adapt to the conditions surrounding the bacterial biofilm. In *S. aureus*, the quorum sensing process is mediated by autoinducing peptides (AIPs) as signaling molecules, with the accessory gene regulator system playing a pivotal role in orchestrating the production of AIPs and various virulence factors. Several quorum inhibitors showed promising anti-virulence and antibiofilm effects that vary in type and function according to the targeted molecule. Disrupting the biofilm architecture and eradicating sessile bacterial cells are crucial steps to prevent colonization on other surfaces or organs. In this context, nanoparticles emerge as efficient carriers for delivering antimicrobial and antibiofilm agents throughout the biofilm architecture. Although metal-based nanoparticles have been previously used in combatting biofilms, its non-degradability and toxicity within the human body presents a real challenge. Therefore, organic nanoparticles in conjunction with quorum inhibitors have been proposed as a promising strategy against biofilms. As nanotherapeutics continue to gain recognition as an antibiofilm strategy, the development of more antibiofilm nanotherapeutics could offer a promising solution to combat biofilm-mediated resistance.

## Methicillin-resistant *Staphylococcus aureus*: the super bug

In 1942, the first instance of penicillin resistance in hospitalized patients was identified among *Staphylococcus aureus (S. aureus)* strains. Subsequently, penicillin resistance became prevalent, with reports indicating its presence in 80% of staphylococcal isolates sourced from both hospital- or healthcare-associated and community-acquired cases [1]. After the widespread emergence of penicillin resistance, methicillin, the first semisynthetic penicillinase-resistant penicillin, was introduced; however, shortly thereafter, a new resistant phenotype known as methicillin-resistant *Staphylococcus aureus* (MRSA) emerged in clinical settings [2]. This resistant phenotype is linked to the production of penicillin-binding protein 2a (PBP2a), which facilitates transpeptidase activity, hence, impeding the treatment of MRSA-mediated infections. MRSA differs genetically from methicillin-sensitive *Staphylococcus aureus* (MSSA) by the presence of the mec gene, encoding a 76 Kda PBP2a [3]. Nowadays, vancomycin can only be given in some cases because of the emergence of vancomycin-resistant *S. aureus* strains (VRSA), which acquired vanA gene clusters [4].

Over the past two decades, a notable shift in the epidemiology of MRSA infections has occurred. Initially confined to healthcare settings, MRSA infections were primarily observed among employees and patients with compromised immune systems, who had been exposed to hospital settings [5]. Shortly thereafter, community-acquired strains (CA-MRSA) emerged, predominantly causing infections in healthy individuals. Due to the effective adaptation of this clone in the host niche, CA-MRSA has extensively proliferated, taking the forefront in generating a rising incidence of unforeseen and invasive healthcare-associated infections [6]. Livestock-associated MRSA (LA-MRSA) evolved through human-to-animal host jumps, via clones that originally infected cattle but could occasionally adapt to infect human [7].

## Biofilm mediated antibiotic resistance in MRSA

Biofilms represent matrix-enclosed sessile communities formed by adherent microbial cells. The formation of biofilms is one of the strategies employed by bacteria to resist the effects of antimicrobial agents, including antibiotics, during infections. For instance, *S. aureus* biofilms decrease the bacterial susceptibility to vancomycin where bacterial biofilms require minimum biofilm inhibitory concentrations; almost 10 times more than the minimum inhibitory concentration (MIC) of vancomycin on planktonic bacteria [8]. Biofilm-mediated resistance to antibiotics is either intrinsic or acquired as detailed below:

### Intrinsic resistance mechanisms

Intrinsic mechanisms of resistance include the biofilm architecture and phenotype. The biofilm matrix is composed of extracellular polymeric substances (EPS) that act as a stabilizing scaffold; enhancing cellular attachment to the colonization site [9]. This matrix can block the access of antibiotics to the vicinity of the biofilm, either by a chemical reaction with the antibiotic, such as deactivation of reactive chlorine species, or by decreasing the rate of diffusion as in oxacillin, vancomycin, and cefotaxime in *S. aureus* and *Staphylococcus epidermidis* (*S. epidermidis*) biofilms [10].

Another intrinsic mechanism of biofilm-mediated resistance involves modified phenotypic features, including gene transcription and growth rates. Slow growth reduces metabolism and diminishes the uptake of antibiotic molecules, providing protection against certain classes like fluoroquinolones, which require internalization. This protective mechanism was demonstrated in *S. epidermidis* biofilms exhibiting resistance to tobramycin [11].

### Acquired resistance mechanisms

Acquired resistance mechanisms involve horizontal gene transfer (HGT) and exchange of plasmid-mediated resistance genes. Plasmids are transferred from one bacterium to the other through conjugation, with higher chances of transfer within bacterial biofilm due to the relatively closer proximity of the cells and the absence of disturbing shear forces, as it provides protection against any interference either in their cell-to-cell contact or their conjugation pili. Therefore, the rates of HGT in biofilms are greater compared to their planktonic counterparts [12].

## Biofilm structure and clinical relevance

Biofilms, formed by microbial cells and EPS, consist of organized architectures with discrete pillar- or mushroom-shaped structures. Such structures form meticulous channel networks acting as nutrient transport systems which connect the biofilm core with the external environment [13]. Nutrient-deficient conditions may trigger biofilm formation. The EPS matrix of the biofilm sequesters any amount of environmental metal nutrients found in the surrounding such as carbon, phosphates, and nitrogen [14]. Bacterial cells within a biofilm enter a quiescent state, known as persister cells, exhibiting reduced metabolic activity, slow rates of cell division, and high tolerance to antimicrobial agents [15]. Upon detachment and dispersion, persister cells regain metabolic activity and antibiotic sensitivity.

Biofilms represent one of the clinically significant virulence factors of *S. aureus* [9]. Moreover, biofilms facilitate the spread of infections by releasing planktonic cells, which can initiate biofilm formation elsewhere. *S. aureus* biofilms can develop inside host tissues or on implanted medical devices such as catheters, cardiac pacemakers, prosthetic joints, contact lenses, cerebrospinal fluid shunts, and prosthetic heart valves [16, 17]. These devices acquire a coating of host matrix proteins before insertion [18]. Since the bacterial cell wall anchors proteins specifically tailored for host matrix proteins, bacterial attachment and anchoring to these medical devices is facilitated. Moreover, *S. aureus* is associated with several chronic biofilm-mediated infections such as endocarditis, nasal infections, cystic fibrosis, urinary tract infections, and skin, and soft tissues infections [19] [18].

Infections mediated by bacterial biofilms pose a serious health hazard as they confer increased resistance to therapeutic interventions. Conventional antibiotic treatments primarily target planktonic cells, leaving biofilm cells capable of detachment and dissemination to other sites for recolonization. Biofilms are bacterial defensive mechanism against host immune responses and clearance, including phagocytosis and protease defenses [9]. Pathways implicated in the structuring and development of biofilms generate protein products with immune-evading functions, affecting both innate and adaptive immune responses through the production of hemolysins, nucleases, proteases, lipases, collagenases, and other degradative enzymes [20]. Evasion from innate immunity includes resistance to phagocytic-mediated killing by neutrophils, escape from neutrophil extracellular traps, inhibition of complement activation, and impairment of macrophage phagocytosis. Conversely, mechanisms of evasion from adaptive immunity mediated by biofilms involve the activation of superantigens and exotoxins, inducing nonspecific T-cell stimulation, and subsequent production of pro-inflammatory cytokines [20].

## Biofilm developmental stages

The development of new and more effective approaches targeting *S. aureus* biofilms requires unraveling the developmental stages underlying the formation of this complex bacterial architecture. Amongst all varying bacterial species, biofilm formation follows a unified model consisting of three main sequential stages; (1) attachment, (2) maturation, and (3) detachment and dispersal. The biofilm is constructed vertically, then it layers and expands horizontally in later stages as a sign of its maturation. The first step of biofilm formation is the attachment and adherence of the bacterial cells to both biotic and abiotic surfaces [21], depending on the generation of compatible attraction forces. In case of biotic surfaces, the attraction force is normally the resultant of a protein-protein interaction. In case of abiotic surfaces, forces such as Van der Waal’s forces, electrostatic forces, or steric interactions mediate attachment of bacteria. As the bacterial cells adhere and proliferate, they form aggregates of micro-colonies, which give the biofilm its mushroom-like structure. These micro-colonies establish the three-dimensional architecture via EPS, which acts as a scaffold allowing layering of cells. The final stage involves cellular detachment and dispersal, initiated when a specific cellular density activates bacterial communication pathways, ultimately leading to the degradation of the EPS. The cells within the biofilm have innate ability to “sense” the surrounding cellular density in a process known as quorum sensing (to be discussed later). This specific density acts as a marker that signifies initiation of cellular detachment through production of proteases, phenol soluble modulins, and nucleases to promote recolonization of distal sites [22].

*S. aureus* biofilm development, as observed through microfluidic flow-cell systems and time-lapse microscopy, encompasses the same three fundamental stages found in other bacterial species, with the addition of two further stages. Yarwood et al. investigated growth and detachment waves correlating with a specific pattern of accessory gene regulator (agr) expression [23]. The identification of the two additional stages in *S. aureus* biofilm formation stemmed from their distinction from agr-mediated dispersal events that occur during maturation and tower formation. Consequently, these stages, labeled as “multiplication” and “exodus,” were positioned among the initial events in the biofilm formation timeline [24]. Detailed information about the molecular mechanisms involved in each stage of biofilm formation is provided in Table 1.

**Table 1:** Genes involved in biofilm development in *S. aureus* categorized by every developmental stage

Gene (Bacterial strain)	Product	Role in biofilm development	Reference
A) Attachment			
srtA (RN4220, OS2 and Newman strains)	Sortase A	Extracellular transpeptidases responsible for anchoring the cell-wall-anchored proteins in *S. aureus* and Gram-positive bacteria.	[25]
fnBP A/B (8325 strain)	Fibronectin-binding protein A and B	Members of the MSCRAMMs group responsible for binding to components of the extracellular matrix such as fibrinogen, fibronectin, and elastin.	[26]
Clf A/B (Newman strain)	Clumping-factor A and B	Glycoprotein; members of the MSCRAMMs group responsible for clumping bacterial cells prior to recognizing and binding to host matrix fibrinogen.	[27]
spA (ISP479r strain)	Protein A	A cell wall-anchored protein recognizing platelet-secreted immunoglobulin G	[28]
EbpS (strain 12,598 Cowan)	Elastin-binding protein	A cell-wall protein responsible for binding to the N-terminal region of the elastin present in host extracellular matrix.	[29]
Pls (1061 strain)	Plasmin-sensitive protein	A virulence factor encoded by a gene carried on the staphylococcal cassette chromosome (SCC) mec type I in MRSA. It stimulates biofilm formation.	[30, 31]
SasG (8325–4 and Newman strains)	*S. aureus* surface protein G	Surface protein that allows attachment to squamous and desquamated epithelial cells, promotes colonization and stimulates biofilm formation.	[32]
IsdA (8325–4 strain)	Iron-regulated surface protein A	Surface protein; part of the NEAT protein family, has a role in binding to fibrinogen, fibronectin, and loricrin.	[33]
Bbp (E514 and O24 strains)	bone sialoprotein-binding protein	Part of the MSCRAMMs; interacts with bone sialoprotein which is a major component of dentine extracellular matrix (ECM) and bones. It plays a significant role in the colonization of bone tissues.	[34]
dltABCD operon (Sa113 wild-type strain)	D-alanylation proteins	Encodes proteins that act synergistically to mediate the D-alanylation of the teichoic acids; wall-teichoic proteins and lipoteichoic acids anchored in the cell wall.	[35]
AltA (8325–4 strain)	Peptidoglycan hydrolase	An enzyme that has been shown to promote binding to hydrophilic and hydrophobic polystyrene surfaces.	[36]
B) Cell-to-cell adhesions and intercellular attachment			
icaABCD operon (SA113 strain)	polysaccharide intracellular adhesin (PIA/PNAG)	Polymeric carbohydrates form complex networks with each other and are anchored to cell surfaces.	[37]
icaR (RN4220 strain)	intercellular adhesin locus regulator	Locus responsible for downregulating the expression of the icaABCD operon.	[38]
SasG (8325–4 and Newman strains)	*S. aureus* surface protein G	Surface protein involved in the protein-mediated ica-independent mechanism of accumulation and cell-to-cell attachment.	[32]
C) Exodus			
AltA (8325–4 strain)	Peptidoglycan hydrolase	AtlA-mediated lysis of the bacterial cells is crucial for the development of the biofilm.	[36]
cidA (UAMS-1)	Putative holin protein	The product of this gene mediates cell lysis during biofilm development.	[39]
Nuc (USA300)	Degradative nucleases	These nucleases play a role in early dispersal of biofilm cells through degrading the eDNA present in the biofilm matrix.	[40]
SaeS (ISP479 and Newman strain)	Sensor histidine kinase	This kinase is responsible for phosphorylation-mediated activation of the SaeR gene upon recognition of environmental signals.	[41]
SaeR (ISP479 and Newman strain)	Response regulator	Upon being activated by the sensor histidine kinase, it induces transcription of around 20 virulence genes	[41]
D) Maturation			
lrgAB (8325–4 strain)	Putative antiholin	Responsible for inhibiting cell death/lysis, typically those initiated by the cidABC operon.	[42]
cidA (UAMS-1)	Putative holin protein	The product of this gene mediates cell lysis and plays a role in biofilm maturation along with the products of the lrgAB operon.	[39]
E) Dispersal			
Aur (USA300 strain)	Zinc-dependent metalloprotease aureolysin	This protein is crucial for pathogenesis as it targets the components of the complement system inside the infected host. It also targets ClfB and Bap.	[43]
SspB (RN4220 strain)	Cysteine protease	Protease involved in the degradation of collagen.	[44]
SspA (RN4220 strain)	Serine protease	Protease involved in the degradation of FnBP and Bap.	[44]
F) Quorum Sensing			
agrA (RN4220 strain)	AgrA response regulator	AgrA is for controlling the genetic adaptation in response to this signal.	[23]
agrC (RN4220 strain)	Transmembrane signal receptor	AgrC is a histidine kinase responsible for the detection of environmental signals.	
agrB (RN4220 strain)	AgrB secretory protein	ArgB is responsible for post-translational modification of AgrD and its secretion into the biofilm milieu.	
agrD (RN4220 strain)	AgrD precursor protein	This precursor protein is processed by the AgrB protein to give a mature autoinducing octapeptide.	
ccpA (SA113)	Catabolite control protein A	CcpA is responsible for regulating gene expression in response to different sugars used as carbon sources.	[45]
Fur (SH1000 and Newman strains)	Ferric uptake regulator	Controls iron concentrations inside bacterial cells and is involved in a complex regulatory network with both Agr and Sae systems	[46]

### Attachment

Attachment and adherence to a surface constitute a crucial initial step that triggers all subsequent stages of biofilm formation. Multiple factors govern the net balance of attraction and repulsion forces between bacterial and polymeric surfaces. These factors encompass the critical distance between the organism and the surface (approximately 1 nm), the characteristics of the surface, whether biotic or abiotic, and the nature of the polymeric substance [47].

Adhesion to biotic surfaces takes place through a group of cell wall-anchored (CWA) proteins specifically compatible with matrix substrates. These include microbial surface components recognizing adhesive matrix molecules (MSCRAMMs) characterized with a common LPXTG motif, a hydrophobic domain, and a tail of positively charged amino acids [48]. The LPXTG motif functions as a sorting signal, guiding the anchorage of proteins into the cell wall [26]. Both the hydrophobic domain and the positively charged tail contribute to retaining the protein in the secretory pathway, where LPXTG is eventually processed and recognized for anchorage. The Sortase family of extracellular transpeptidases is responsible for cleaving the LPXTG motif and catalyzing its insertion into the cell wall. In *S. aureus* and most Gram-positive bacteria, recognition, and cleavage of the LPXTG motif are mediated by the srtA gene product, known as sortase A. Sortase A facilitates anchorage by transpeptidation to the peptidoglycan of the cell wall, followed by LPXTG cleavage and attachment of the protein to the pentaglycine cross-linker within the peptidoglycan [26]. *S. aureus* possesses up to 21 different LPXTG-containing proteins displayed on its surface. These proteins are processed and cleaved at their LPXTG motif by sortase A. Unlike their shared motifs, these microbial surface components have specific binding domains that target certain components of the matrix. To start, fibronectin-binding proteins A and B can bind to the γ domain of fibrinogen through their C-terminus, to elastin via their A domain, or to fibronectin via their fibronectin-binding domains [49–51]. The clumping factors ClfA and ClfB are also members of the MSCRAMMs group which can recognize and bind to host matrix fibrinogen [27]. These fibrinogen receptors are known as clumping factors due to the clumping of bacterial cells before interaction with fibrinogen. ClfA protein binds to the γ chain of fibrinogen, while ClfB binds to α- and β-chains [52]. There are also receptors for collagen protein on the surface of *S. aureus*. These collagen binding MSCRAMMs are called Collagen-binding adhesin (CNA). The collagen-binding domain of the CNA is found on the A-region of the protein. The binding mechanism does not depend on the presence of metal ions. However, it occurs via a “hugging” mechanism in which the two subdomains of the CNA surround and wrap the collagen monomer. Protein A (SpA) is also a cell wall-anchored protein recognizing platelet-secreted immunoglobulin G and von Willebrand factor, playing a significant role in infections such as endocarditis [28]. Other MSCRAMMs include elastin-binding protein [51], plasmin-sensitive protein [30], SasG [53], iron regulated surface determinants IsdA/B/C/H [33], and bone sialoprotein Bbp [54].

There are other CWA-independent mechanisms of attachment that may contribute to bacterial adherence to abiotic surfaces. Studies have shown the involvement of these mechanisms in facilitating the binding of bacterial cells to polystyrene. One of these mechanisms is mediated by the negatively charged teichoic acid polymers integrated into the bacterial cell wall. Teichoic acids constitute significant components of the Gram-positive bacterial cell wall, accounting for 60% of the total mass. They are either stabilized in the plasma membrane as lipoteichoic acids or anchored in the cell wall through covalent bonds to the peptidoglycans, forming wall teichoic acids [55]. The principle of binding depends on the attraction force between the cell wall and the attachment surface (polystyrene or glass) [56]. The physicochemical properties of the cell wall depend on the teichoic acid polymers. Teichoic acids in the *S. aureus* cell wall consist of alternating phosphate and ribitol units and contain a higher number of negatively charged phosphate groups compared to the D-alanine residues. Teichoic acids enable bacteria to attach to hydrophobic surfaces or surfaces that are slightly negatively charged. This adhesion is facilitated by attractive van der Waals forces and interionic forces, where the latter can be either repulsive or attractive. The bacterial ability to attach depends on the resultant net force. This was demonstrated by using *ΔdltA* *S. aureus* mutants, where the *dltA* gene is responsible for incorporating the D-alanine in teichoic acids. These mutants are characterized by increased negativity of the cell wall, consequently, the repulsive forces overcome the attraction forces and prevent adherence to the plastic surface [56]. Another mechanism involves the autolysin enzyme AtlA. *S. aureus* strains demonstrating clustering capabilities exhibited a biofilm-negative phenotype upon *AtlA* deletions [57]. Atl is a wall-anchored peptidoglycan hydrolase found in staphylococci species, with AtlA hydrolase specifically identified in *S. aureus* strains. This enzyme has been demonstrated to facilitate binding to both hydrophilic and hydrophobic polystyrene surfaces [36, 57]. *S. aureus* can also employ CWA proteins in its attachment to abiotic surfaces. In this scenario, it is crucial for the implanted device to be covered by host matrix proteins before attachment. Several studies have demonstrated the dependence of MRSA on the Atl-FnBP biofilm phenotype [36].

### Multiplication

The multiplication stage of biofilm development is a stabilization phase that reinforces the initial attachment, hence protecting the first horizontal layer of cells from being removed by the shear forces of flowing fluid. The key players in this phase are factors directed towards promoting cell-to-cell adhesions and intercellular attachment. In biofilms, the bacterial cells constitute a small portion (only around 10%) of the dry mass. The remaining portion, totaling about 90%, serves as the stabilizing scaffold that “fixes” the cells in place to allow intracellular attachment. This scaffold, known as EPS, is heterogeneous, and is composed of various biopolymeric substances that impart a “sticky” nature suitable for its function [54]. The EPS represents a sophisticated relationship between composition, structure, and function. Each component has a specific function, not only during formation but throughout maturation and dispersal as well. The EPS is composed of exopolysaccharides, exoproteins, extracellular DNA, surfactants, lipids, and water, each in a specific ratio, and it serves various functions, including adhesion, cellular accumulation, sorption of organic and inorganic molecules, and water retention. Moreover, it acts as a protective barrier, a source of nutrients, an electron donor/acceptor, and a sink for excess energy [58]. Staphylococcal biofilms are classified according to the major component of the EPS into either polysaccharide matrices or proteinaceous matrices.

The exopolysaccharides constitute the major component of biofilms with polysaccharide matrices. These polymers can be found in branched or linearized forms, with molecular masses ranging from 0.5 to 2 × 10^6^ Da [58]. These polymeric carbohydrates form complex networks with each other and are anchored to cell surfaces. Exopolysaccharides are not specific to the environment in which the biofilm is formed, as they are detected in biofilms isolated from pure cultures, soil, water systems, and human chronic infections. However, these polysaccharides differ from one bacterial species to another in the type of monomers involved in polymerization. Homopolysaccharides, such as glucans, fructans, and cellulose, are found in streptococcal biofilms and biofilms produced by Pseudomonadaceae and Enterobacterales. EPS are also found in heteropolysaccharide form, where the monomers are a collection of neutral and charged residues. For *S. aureus*, polysaccharides found in the EPS matrix are usually polycationic. An example is the polysaccharide intracellular adhesin PIA/PNAG, which is a polymer made up of β-1,6-linked N-acetylglucosamine with partially deacetylated residues [58]. This polymer facilitates colonization of implanted devices and plays a role in chronic infections. PIA is the major constituent of the exopolysaccharide staphylococcal biofilm matrix in strains with activated ica operon. The intercellular adhesion *ica* locus is the operon responsible for encoding the PIA. It was initially identified as a detection marker for the biofilm-formation phenotype in *S. epidermidis* strains [59] and was later identified in *S. aureus*. It is part of the “accessory-genome” rather than the “core-genome” of the bacteria, making it more common among clinical strains, and potentially absent in others. In early studies, the ica locus was detected in strains isolated from implant-associated infections [60]. It is composed of four different genes (*icaA*, *icaB*, *icaC*, and *icaD*), collectively producing PIA. The *icaA* and *icaD* genes are involved in exopolysaccharide synthesis. The *icaA* gene encodes a transmembrane transferase enzyme responsible for the production of the poly-N-acetylglucosamine polymer. The activity of this enzyme is dependent on its co-expression with the *icaD* gene product to produce enzymatically active peptides longer than 20 amino acid residues [61]. The icaC protein is the ica-system component responsible for translocating the poly-N-acetylglucosamine polymer to the cell surface. Finally, the last acting protein is the product of the *icaB* gene, which deacetylates the polymer. The deacetlyation step is the fixative step signifying development of the biofilm exopolysaccharide by anchoring the polymer to the outer surface of the bacterium [62]. The expression of the *ica* locus is negatively regulated by the intercellular adhesin locus regulator *icaR* gene, which acts under the influence of SarA protein (later discussed in quorum-sensing) and stress sigma σ^B^ [63]. The *ica* locus can be regulated by the phase-variation property of virulence factors, where the expression can be turned on and off depending on the environment, and in cases of evasion. In *S. aureus*, phase-variation is carried out by the expansion or contraction of a 4-nucleotide tandem “TTTA” repeat in the *icaC* gene, leading to a frame-shift mutation and consequently resulting in a truncated, non-functional icaC protein [64]. The inactivation of PIA/PNAG production is thought to contribute to infection and bacterial fitness.

Exoproteins are also present as a core constituent of the EPS in all types of matrices. These proteins have functional roles in attachment, structure, and degradation. The role of attachment proteins is particularly dominant in proteinaceous matrices, and contributes to strengthening the initial attachment. These matrices are more commonly found in MRSA isolates. They are ica-independent, however, the *ica* operon is present in the genome. It is rendered inactive and does not express the *icaABCD* genes. Nevertheless, the discovery of the ica-independent pathways require deletion of the *ica* locus, since mutated strains were able to propagate and form biofilm, regardless of their compromised PIA production [65]. For *S. aureus*, the Bap protein was identified as the main player in surface attachment and intracellular adherence [66]. However, it was later discovered that the ica-independent mechanisms are multifactorial and involve varying components that allow cell-to-cell adhesion without production of extracellular polysaccharide. The protein nature of these matrices was concluded upon observing the effect of proteases on ica-independent biofilms [67]. Cells in ica-independent biofilms form aggregates with assistance of fibronectin-binding proteins and other MSCRAMMs proteins, such as FnBPs, ClfB, and SdrC. The role of these MSCRAMMs proteins extends beyond initial attachment, as they are implicated in mechanisms that keep the cells in close proximity with each other in the absence of an extracellular amorphous matrix. They have dual function mediating both attachment and accumulation in biofilm development [68]. Several other proteins are also implicated in the process such as Protein A [28], SasC [69], and SasG [32]. The protein-mediated ica-independent accumulation mechanism can be exemplified by the action of the SasG protein; a surface protein found in *S. aureus*. SasG protein encompasses N-terminal A domain and repeated B domains. Initially, the SasG is anchored into the cell wall, then it is cleaved at its B region. Following this, the fragmented SasG is released into the exterior, and acts to dimerize with exposed B domains on other bacterial cell surfaces in a non-covalent manner in the presence of Zn^2+^. The fragmented and exposed SasG B regions on adjacent neighboring cells interact with each other leading to cell accumulation and biofilm formation [32]. The ica-independent biofilm formation mechanisms indicate that production of PIA is considerably strain- or condition-specific. All *S. aureus* clinical strains harbor the *ica* locus [70]. Therefore, the alternative pathways identified can be interpreted as environmentally induced phenomena, as they are “switched on” to adapt the biofilm characteristics to an external stimulus.

Another mode of bacterial cell accumulation involves utilizing cytoplasmic proteins as matrix components. These cytoplasmic proteins are released from the cells during the stationary phase and associate with the cell surface. This represents a form of “recycling” mechanism during biofilm accumulation, where cytoplasmic proteins aggregate in the interstitial space between neighboring cells under the influence of biofilm-inducing conditions, such as low pH. Enolase and GAPDH are examples of such “moonlighting” cytoplasmic proteins that act as matrix components [71]. These proteins lack signal peptides that dictate their translocation into the external milieu. However, it has been suggested that they contribute to biofilm accumulation similarly to extracellular DNA (eDNA). Through regulated autolysis, enolase, GAPDH, and other proteins such as phenol-soluble modulin (PSMs), beta-toxin (Hlb), and cytoplasmic nucleoid-associated proteins bind to eDNA and act externally to stabilize the extracellular matrix [72–74]. In addition to the aforementioned proteins, the EPS can also include degradative enzymes that are released during the dispersal stage of biofilm.

eDNA is an integral part of the EPS and was discovered using *S. aureus* mutants with a defective autolysin protein, AtlA. The autolysin AtlA is a bifunctional enzyme that is cleaved to yield two catalytically active proteins; amidase and glucosaminidase. AtlA-mediated lysis of bacterial cells is crucial for the development of the biofilm, as mutants exhibit a reduced formation phenotype [75]. The significance of eDNA and cellular lysis is emphasized in the subsequent exodus stage.

### Exodus

Exodus is the third stage of biofilm formation observed after 6 hours from initiation. This stage is considered an early dispersal phase independent of agr-mediated dispersal, contributing to the reconstruction of the biofilm structure. It involves a self-regulated nuclease-dependent degradation of the eDNA found in the matrix. eDNA is a major component in the structure of the *S. aureus* biofilm; released into the milieu from lysed bacterial cells. Studies have reported the involvement of *cidA*; a gene regulated by the CidR regulator acting on the *cidABC* operon, in controlling cell lysis during biofilm development [76]. The *cidA* mutants produced biofilms with altered appearance and more roughness [39]. Furthermore, the significant role of eDNA in the integrity and structuring of biofilms, specifically those of *S. aureus*, was discovered upon treating *S. aureus* biofilms with DNase I. DNase I inhibited biofilm formation, resulted in the detachment of preformed biofilms, and increased susceptibility to detergents [76]. This further demonstrates the importance of *cidA *and eDNA in the formation of biofilms [77]. The function of the nucleases released prior to the exodus event can be correlated with the significant reduction in the biomass of the *S. aureus* biofilm [78]. Only a subpopulation of cells can express their *nuc* gene and produce the nucleases to degrade the eDNA, allowing the detachment of most of the accumulated population. The expression of the *nuc* gene and production of the degradative nucleases are processes tightly regulated by a two-component system known as Sae (*S. aureus* exoprotein expression). Two-component systems are bacterial signal transduction pathways that allow bacterial survival and adaptation in the environment. These pathways mediate processes of signaling, transduction, and transcriptional activation using only two proteins, which can detect environmental signals and stimulate the genetic adaptation of bacteria [79]. The Sae two-component system is also composed of two proteins; SaeS sensor histidine kinase and the SaeR response regulator [80]. The SaeS sensor histidine kinase recognizes environmental signals, such as human neutrophil peptides, or human α-defensins, and through a cascade of phosphorylation events, it activates the effector regulatory molecule, SaeR, and induces transcription of approximately 20 virulence genes [80].

The biological significance of the exodus stage can be attributed to its implication prior to biofilm tower formation. The reduced biomass is suggested to be a prerequisite to produce secondary structures in the biofilm architecture. Studies showed that *nuc* mutants of *S. aureus* do not have an exodus stage during their biofilm production, and are consequently unable to form microcolonies, which are crucial for the subsequent maturation stage [24].

For the last two stages, maturation and dispersal, the difference in the biological importance of each stage must be stressed to compensate for the complexity resulting from the intertwining mechanisms that are shared between them. Both stages involve a degree of dispersal and detachment, where cells are released from the biofilm structure into the surrounding vicinity. In the maturation stage, the dispersive processes are complemented with adhesive processes, resulting from the involvement of these dispersive processes in the architectural reconstruction of the biofilm, as it is reorganized into its final “mature” form that ensures stability and viability of the cells within. On the other hand, the dispersal stage involves release of cells for distal recolonization and spreading of the biofilm phenotype. Accordingly, the detachment process is more pronounced.

### Maturation

The maturation stage is a phase of continued cellular growth that results in the development of bacterial colonies consisting of millions of cells tightly organized into three-dimensional mushroom-shaped masses [24]. A distinctive feature of the maturation stage is microcolony formation. These microcolonies create additional surface areas that facilitate the exchange of nutrients, which are crucial for the viability of the three-dimensional tower structure. During this stage, there is an interplay between adhesive and disruptive processes that act to restructure the biofilm; carving out open water channels for the exchange of nutrients and waste products. This allows the biofilm to expand and grow while maintaining the transport of nutrients to the internalized population of cells found at a distance from the surface.

**Fig. 1:**
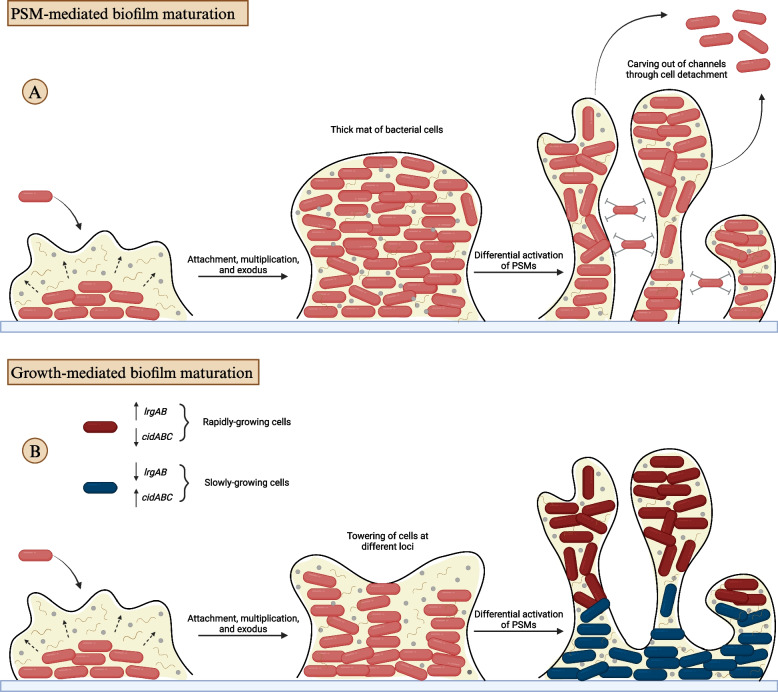
Models proposed to illustrate biofilm maturation. **A** PSM-mediated biofilm maturation model. **B** Growth-mediated biofilm maturation model

There are two models that describe the formation of biofilm microcolonies (Fig. 1). The initial model explains the carving out of channels using the functional role of phenol-soluble modulins (PSMs) in cellular dispersal. PSMs are amphipathic in nature with surfactant-like properties; allowing the disruption of non-covalent interactions between matrix components [78, 81]. The model proposes that biofilm maturation and microcolony formation are subtractive processes, where the thick mat of biofilm bacterial cells is produced first, and then PSMs regulate detachment and dispersal of cells from specific locations, leading to the emergence of fluid-filled channels. This model depends on variations in the expression of PSMs at different locations in the biofilm, controlled by differences in the activity of the agr quorum-sensing system [81]. However, the use of time-lapse microscopy provided insights into the actual mechanisms of the process, leading to the development of a different biofilm maturation model [82].

The second model proposes that microcolonies grow at different foci of cells that remain at the basal layer of the biofilm, attached to the surface shortly after the onset of the exodus stage. The study also demonstrated a differential pattern of metabolism among the microcolony subtypes present within the same biofilm. This pattern manifested as variations in growth rates, where rapidly growing microcolonies extended from slower-growing cells at the basal layer. Such variations resulted from differences in the expression of the *cidABC* and *lrgAB* cell death-associated operons, with rapid growth associated with increased constitutive expression of *lrgAB*, and slower growth associated with constitutive *cidABC* expression and no detectable *lrgAB* expression. These two models are not contradictory, and do not necessarily replace one another. The patterns of PSM expression potentially play a role in the reconstruction of the biofilm structure in collaboration with the other proposed mechanism for the formation of microcolonies. However, the existence of both models can be attributed to the involvement of PSMs in the dispersal stage, indicating a time-dependent implementation of these models where PSMs act at a later point. Nevertheless, the observations made by Moormeier and his group revealed an additional biological significance to the maturation stage of biofilm formation. It enables the metabolic diversification of the microcolonies within the biofilm, allowing for its persistence against inevitable environmental stresses that endanger its existence. This proposed “programming” process gives rise to cells with varying metabolic activities, despite emerging from a common, genetically identical background. This diversification reduces the time needed for adaptation to stressors and enhances resistance to antimicrobial agents. However, in cases where the stress becomes intolerable, the biofilm dispersal mechanisms ensure survival in other more habitable sites [83].

### Dispersal

EPS is heterogeneous, comprising proteins, eDNA, and polysaccharides. Degrading the biofilm EPS matrix necessitates an array of distinct degradative exoenzymes; each targeting a different component. The proteinaceous component of biofilms is vulnerable to degradative proteases that play a key role in mediating biofilm dispersal. *S. aureus* has the ability to synthesize and secrete 10 different proteases, each with prominent roles in cellular detachment. Examples of such proteases are the zinc-dependent metalloprotease aureolysin [84], the two cysteine proteases SspB and ScpA [20], and seven serine proteases known as SspA and SplA to -F [44, 85]. Each protease targets specific proteins involved in cellular attachment. For instance, the serine protease SspA acts on FnBP and Bap proteins, which are implicated in initial attachment and intracellular adhesion [66, 86]. The Aur protease acts on the Bap protein as well, however, it also targets the ClfB protein instead of the FnBP [87]. The expression of these proteases is regulated by various transcriptional regulators, such as SigB, Rot, SarA, and SaeRS [78]. These regulation mechanisms of the exoenzymes responsible for biofilm dispersal are all agr-independent mechanisms. However, the quorum-sensing agr system plays a central role in the dispersion process by regulating both exoproteases and PSMs; both contributing to cell detachment from the matrix. This mechanism is discussed in the following section.

## Role of quorum sensing in *S. aureus* biofilm formation

Quorum sensing (QS) is a process in which each bacterium can “sense” the number of surrounding bacterial cells. This sensing mechanism enables a coordinated response to the surrounding environment by regulating gene expression patterns within the community [88]. Depending on cell density, bacterial cells can produce, detect, and respond to specific extracellular signaling molecules known as autoinducers, which serve as mediators of the QS phenomenon. Autoinducers are self-coded, meaning they are produced and released by the same bacterium that demonstrates their effect. Additionally, they are stage-specific, with production and detection induced by a specific growth stage or abrupt environmental change. To be considered an autoinducer, a molecule must accumulate extracellularly and be recognized by bacterial surface receptors. The extracellular accumulation must also trigger a cascade of cellular signaling within the cell, not resulting from the metabolization or detoxification of the molecule [88].

Autoinducers can either be auto-activators or autocrine regulators. Auto-activators promote the expression of their genes, upregulating their own synthesis in a positive feedback loop. Accordingly, once the production of auto-activators is initiated, it prompts an exponential increase in their concentration. On the other hand, autocrine regulators are also signaling molecules involved in QS and the regulation of gene expression; however, they do not upregulate their own synthesis [89].

The nature of autoinducers differs between Gram-positive and Gram-negative bacteria. In Gram-negative bacteria, the released autoinducers are N-acyl-l-homoserine lactones (AHLs), while in Gram-positive bacteria, the autoinducers are peptides (AIPs). AHLs and AIPs differ in their mechanism of action, despite causing the same outcome, which is genetic regulation [89].

AHLs are diffusible molecules that easily traverse the membrane and enter the cell. Inside the cell, they bind to specific intracellular receptor proteins. This binding induces a conformational change in these receptors, causing allosteric unfolding and leading to the formation of dimers. These receptor dimers act as transcriptional activators, thereby activating the regulated genes. In contrast, Gram-positive bacteria require polytopic transmembrane receptors for AIPs, since they cannot easily penetrate the cell wall. The binding of AIPs to these transmembrane receptors initiates signal transduction pathways that modulate gene expression [89].

*S. aureus* employs AIPs in a major QS system known as the accessory gene regulator (agr) system, which encodes a signaling circuit responsible for the production and signaling of autoinducers. The sequence of cascades regulated by the agr system is directly correlated with the function and role of quorum sensing in the regulation of virulence factors during *S. aureus* infection and biofilm formation [90].

During the early stages of infection, the agr system is inactive, allowing bacterial cells to colonize host tissues using the adhesion proteins expressed on their surface. For biofilm formation, these adhesins are crucial for surface attachment, representing the initial step in biofilm formation. Upon attachment along with increasing cellular density, the concentration of secreted autoinducers reaches the activation threshold. These molecules then bind to components of the agr system on the bacterium’s surface, leading to the activation of signaling cascades [90].

The last step of biofilm formation involves cellular detachment and dispersion to facilitate infection and colonization of other sites. Consequently, the incidence of agr system activation coincides with the need for dispersion due to the system’s ability to reduce the production of adhesion factors [90]. The expression of the genes in the agr operon regulates bacterial virulence by decreasing the production of adhesion factors and inducing the production of exotoxins and degradative exoproteins.

The agr locus includes two divergent promoters, P2 and P3. The P2 promoter is part of the *agrABCD* operon, which consists of four genes (*agrA*, *agrB*, *agrC*, and *agrD*) acting together to modulate the expression of the agr locus. The *agrA* and *agrC* genes encode a “density-sensing cassette,” while the other two genes, *agrB* and *agrD*, encode its inducer. The *agrD* gene encodes a precursor peptide known as AgrD. This 46-amino acid peptide chain undergoes post-translational modification and is exported by the AgrB secretory protein located on the cytoplasmic membrane [91]. The modification process is crucial for maturation of the AgrD precursor peptide into an autoinducing octapeptide which possesses a thiolactone ring that is crucial for its signaling activity [92].

In the presence of sufficient amounts of these autoinducing octapeptides, these molecules bind to a transmembrane signal receptor encoded by the *agrC* gene. This receptor is part of a classic two-component signal transduction pathway. The two-component system (TCS) pathway associated with the agr system relies on two proteins; AgrC and AgrA. AgrC detects environmental signals, while AgrA is responsible for controlling genetic adaptation in response to these signals. The binding of the autoinducing octapeptide induces two subsequent phosphorylation events by AgrC. The AgrC histidine kinase undergoes autophosphorylation upon binding to the autoinducing octapeptide, and then it phosphorylates the AgrA molecule, thereby activating it. The activated AgrA functions as a response regulator, acting as a transcription factor by binding to DNA sequences and promoting the expression of adjacent genes. Similarly, AgrA binds to sequences in the P2 and P3 promoter regions. However, the upregulation of the expression of their genes requires simultaneous binding of AgrA and another regulator protein known as SarA [93]. Together, these proteins mediate a positive feedback loop involving the *agrABCD* operon [91]. AgrA protein is also transcribed by the weak constitutive promoter P1. The P1 promoter is found inside the *agrABCD* operon. However, it transcribes AgrA protein only. This weak promoter P1 is also influenced by the binding of the AgrA protein, as the expression is relatively upregulated with increased levels of agrA. Accordingly, agrA can upregulate its own expression from two different sources, through P1 and P2. Further implications of the P1 expression in the agr signaling pathway are yet to be discovered [94].

The Staphylococcal agr system influences over 70 different genes, with 23 identified as virulence factors. This regulatory mechanism involves a regulatory RNA molecule that mediates the circuit, specified by the 514-nucleotide transcript of the P3 operon. The P3 promoter guides the transcription of the RNAIII molecule; the primary effector in the agr signaling pathway, which modulates the expression of two distinct classes of virulence factors.

The first class encompasses cell wall-associated factors such as coagulases, oligopeptides, adhesins, and permeases, controlling attachment to host cells and evasion of the immune system. The second class comprises secreted exoproteins, including proteases, lipases, alpha-, beta-, and gamma-hemolysins, toxic shock syndrome toxins, and superantigens. These are categorized into degradative enzymes facilitating cell invasion and bacterial toxins causing diseases [95]. The RNAIII molecule can also act as a transcript for these exoproteins, where it encodes for δ-haemolysin protein [96]. Each class of these virulence factors is upregulated during different phases of bacterial growth. During the log and exponential phases, the production of cell wall-associated factors increases. However, during the late exponential phase and stationary phase, the expression of cell wall-associated factors decreases while the expression of exoproteins increases, which allows bacterial detachment from the initial colonization site. In case of biofilms, discrete patches of bacterial cells detach from mature biofilms to spread and colonize a new site [97].

Examples of virulence factors regulated by the agr system include the PSM family of staphylococcal δ–toxin [98]. PSMs are key factors in *S. aureus* pathogenicity and are encoded at three different conserved locations in the genome in different operons. Each operon codes for a specific group of PSMs, classified according to the length of the peptide. PSMα peptides, PSMα1–PSMα4, are transcribed from the *psmα* operon. These α-type peptides consist of 20–25 amino acids. PSMβ1 and 2 peptides, transcribed from the *psmβ* operon, are longer, consisting of 44 amino acids. Finally, the δ-toxin shares the same length as the α-type peptides; however, they are found within the coding sequence of the RNAIII of the agr system. The AgrA molecule is responsible for upregulating its own expression, while the actual effector of this pathway is the RNAIII molecule [99]. Nevertheless, expression of the PSMs operons is the only known and confirmed exception that is influenced by binding of AgrA. The *psmα* and *psmβ*, and *psm-mec* gene are all under the influence of AgrA. This agr-dependent modulation of PSMs allows cytolytic killing of neutrophils upon phagocytosis of the bacteria [100]. The phagocytosed bacteria exhibit high levels of agr expression, which is an example of QS induction as a response to environmental stressors. The bacterial cells can “switch on” a more virulent phenotype inside the host neutrophils to allow evasion. This behavior is also observed in biofilm development. PSMs are essential to the structuring of biofilm through their ability to act as surfactants. They allow formation of channel-containing biofilm structures [81], and form fibril-like structures that facilitate biofilm cell accumulation [72]. Furthermore, the link between activation of agr system and PSMs expression links the PSMs with the detachment phase of biofilms [101].

The intraspecies variation of the agr system originates from the variability in the region controlling the specificity of signal processing and the ligand-receptor interactions, covering the main body of AgrB, AgrD, and the sensor domain of AgrC. This variability results from amino-acid polymorphisms, providing specificity to the agr molecules involved in the signaling process. It is demonstrated in variants of AgrD produced, AgrB that processes the peptide, and AgrC that recognizes this Autoinducing Peptide (AIP) [102]. *S. aureus* strains carrying variants of the agr locus have been noted to possess identical conserved and structural genes, excluding mobile genetic elements, which suggests that the variation occurs at the subspecies level. Accordingly, strains with different agr loci are considered different pherotypes [103]. Currently, there are four allelic variants of the agr locus.

**Fig. 2:**
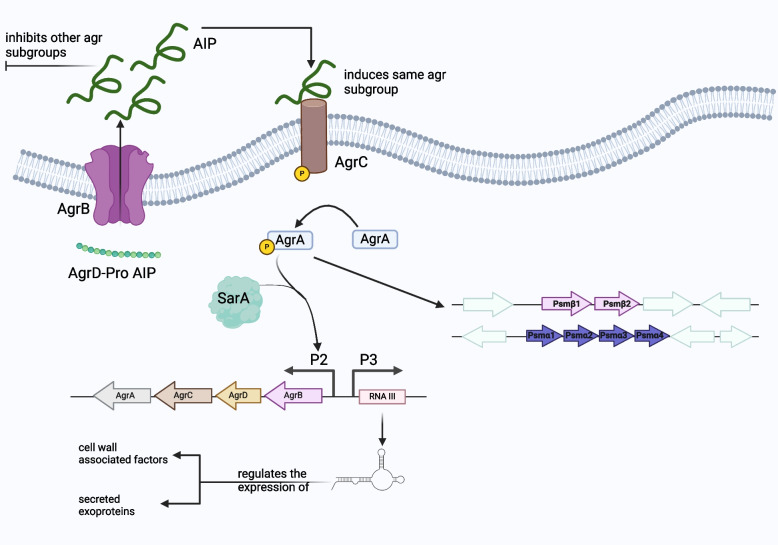
Schematic diagram illustrating the Staphylococcal accessory gene regulatory (agr) system and its role in producing AIP, which contributes to biofilm formation

**Fig. 3:**
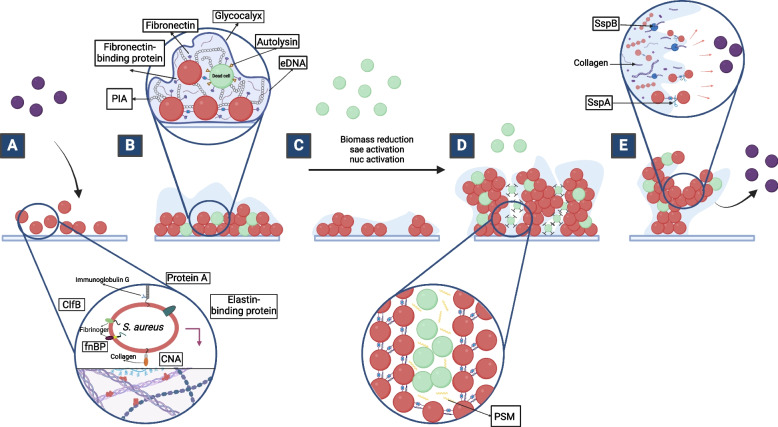
Schematic illustration of biofilm regulatory networks of *S. aureus*

Intraspecies variation plays a significant role in bacterial interference. It has been observed that the AIPs produced by a certain pherotype activating *agr* transcription can inhibit another strain from a different subgroup. For example, AIP-I produced by *S. aureus* belonging to group-I inhibits agr expression in group-II strains. The same principle applies interchangeably among all groups, except for interactions between group I and group IV, which are closely related. These two groups have only one different amino acid residue out of eight residues, allowing them to activate each other but with relatively weak efficacy [104]. Consequently, the bacterial interference phenomenon is particularly prominent during infections with more than one pherotype. The inhibitory role of cross-interactions of AIPs translates to group-based preferences for infection sites and distinct disease patterns. Several studies aimed to establish a relationship between the agr group and the type of disease Figs. 2, 3. For instance, using multi-locus enzyme electrophoresis and pulsed-field gel electrophoresis, the production of toxic shock syndrome toxin-1 (TSST-1) and manifestations of menstrual toxic shock were both attributed to strains harboring group-III agr [104]. Furthermore, the majority of strains producing exfoliatin toxin causing staphylococcal scalded-skin syndrome belong to group-IV [105].

Since the agr system is the main modulator of such a process, it is logical to assume an interactive relationship between the biofilm and the environment in which the agr system is influenced by surrounding cues. These cues can be physiochemical factors such as pH and temperature, or simply the nutritional composition of the environmental niche. Such parameters determine the subsequent profiles of bacterial gene transcription by either downregulating or upregulating certain components of the agr system [78]. One of the earlier relationships developed was the influence of glucose on the agr system [106]. Studies revealed that decreasing levels of pH, as a result of glucose fermentation, significantly inhibited the expression of the agr genes [107]. Accordingly, reports studying the effect of glucose depletion in *S. aureus* biofilm niche delineated the upregulation of the agr system, and the subsequent detachment of bacterial cells to promote dispersal. The linking molecule that translates the presence or absence of glucose into patterns of transcription is the catabolite control protein A (CcpA) [45]. CcpA is responsible for regulating gene expression in response to different sugars used as carbon sources. The *ccpA* gene knockout hindered the ability of the bacteria to accumulate and aggregate without affecting the initial stages of attachment. Regarding other molecules involved in biofilm formation, CcpA was shown to upregulate the expression of *cidA*, involved in the release of eDNA, icaA, and the production of PIA. Therefore, it can be inferred that the presence of glucose upregulates CcpA, which consequently increases the expression of genes required for biofilm formation and aggregation. This justifies the necessity of adding a carbon source (either glucose or sucrose) in the media used for *S. aureus* biofilm formation. Therefore, it can be concluded that the absence of glucose drives the bacterial biofilm to disperse via the action of the agr system, to seek another niche that better supports its growth and metabolism.

In addition to sugars, bacteria depend on metals as micronutrients, integrated into metabolic processes as co-factors. Consequently, their concentration in the surrounding environment can alter the course of gene expression [78]. Iron is one of the metals required for bacterial metabolism, and different types of bacteria have developed mechanisms to acquire iron from the surrounding environment, either in vivo or in vitro. Accordingly, iron-limiting conditions are known to trigger bacterial virulence and the expression of various virulence factors.

*S. aureus* has adapted to consider low concentrations of iron as a major signal cue that induces the expression of virulence factors for iron acquisition, surface adhesion, and biofilm formation, which areall responses to threatening stress conditions [106]. The ferric uptake regulator (Fur) controls iron concentrations inside bacterial cells, and is involved in a complex regulatory network with both Agr and Sae systems. Together, they control the expression of the extracellular adhesion protein (Eap) and the extracellular matrix protein-binding protein (Emp). Both Eap and Emp are secreted proteins with roles in promoting adhesion to host proteins, and Eap additionally functions in immune evasion and bacterial internalization into host cells [46].

As a central component of the regulatory network, Fur induces the expression of Sae under low iron conditions. Sae is essential for biofilm formation [108], immune evasion, response to alpha defensins, response to oxidative stress, and survival in neutrophils [109, 110]. Accordingly, Sae is an important virulence factor of *S. aureus*. This provides further justification for choosing low-iron culture media for culturing *S. aureus* bacteria in biofilm formation assays, as opposed to other iron-rich media such as tryptic soy broth [111]. The first link connecting both Fur and Sae was established by Johnson et al. in 2010 [108], which expands the role of the Fur system from iron acquisition to the regulation of virulence. Fur upregulates the expression of Sae, and it was also demonstrated to upregulate the expression of both Agr and Rot. Agr, on the other hand, downregulates the expression of Fur, yet upregulates that of Sae. This sheds light on the complex regulatory feedback loop involving all three components, mainly influenced by the surrounding levels of iron.

**Fig. 4:**
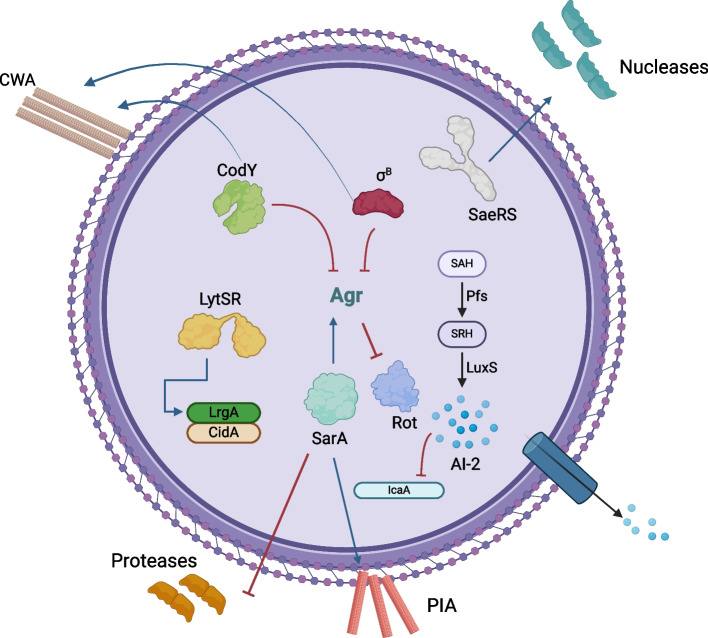
Proteins implicated in the different stages of *S. aureus* biofilm development. **A** Attachment, (**B**) Multiplication, (**C**) Exodus, (**D**) Maturation, and (**E**) Dispersal

There are other physiochemical factors that control QS on a non-genetic level, such as flow rate. Interestingly, high levels of flow rate promote biofilm formation through the wide dissemination of AIPs. Shear forces produced from a high flow rate can transport the signaling molecules in cells found downstream of the biofilm. Hence, these cells may become activated, and induced to form biofilms [112] Fig. 4.

## Biofilm regulatory networks of *S. aureus*

The regulatory networks overlapping with the agr QS system are only some of the networks controlling the process of biofilm formation. *S. aureus* fine-tunes biofilm formation through the integration of several regulatory molecules to intricately couple its biofilm with changing environmental conditions. The complexity of such network allows the bacteria to accommodate any changes and adapt rapidly and effectively. This review has discussed several examples of these regulatory networks, such as the SarA protein family and the Sae two-component system, in relevant contexts. The following section will explore additional examples to provide a full, coherent, and inclusive picture that allows a thorough understanding of *S. aureus* biofilm formation.

### GTP-sensing transcription repressor CodY

CodY is a transcriptional repressor that responds to the availability of nutrients and the metabolic capacity of the cell. Under normal conditions, where the cell has a sufficient supply of nutrients for survival, CodY is associated with its effector molecules. This association increases CodY’s ability to bind to DNA and interfere with RNA polymerase binding and mRNA transcription. However, when the level of nutrients decreases, intracellular levels of GTP and branched-chain amino acids (e.g., valine, isoleucine, and leucine which promote protein synthesis) decrease. This decrease in nutrients reduces CodY DNA-binding affinity, leading to the activation of CodY-repressed genes. Since nutrient-limiting conditions constitute a stress factor on bacterial cells, decreased CodY DNA-binding affinity leads to the activation of the agr locus [113]. It directly binds to the *agrC *gene, which is the signal-sensor component of the Agr-system [113, 114]. *RsaD*, encoding a small regulatory RNA, is another gene that is repressed under the action of the CodY repressor. The *rsaD* sRNA regulates the process of cell death under conditions of acidic stress. It eventually contributes to the release of eDNA and, consequently, biofilm formation [115]. Therefore, under low concentrations of nutrients, bacteria tend to demonstrate higher levels of virulence through the expression of the RNAIII regulatory molecule, transcription of *rsaD*, and mediation of biofilm formation.

CodY is also linked to the repression of polysaccharide intercellular adhesin (PIA) production [38]. Acting directly on the DNA, the CodY-dependent decrease in PIA production is attributed to decreased levels of icaA transcription. As previously mentioned, the *icaADBC* operon can be regulated by other regulatory molecules such as icaR and SarA. However, CodY-mediated repression is independent of these mechanisms. Moreover, CodY controls the repressed expression of the *nuc* gene and extracellular proteases, both of which are considered important modulators of biofilm formation [116], and RNAIII [113]. However, CodY-mediated repression is independent of these mechanisms. Moreover, CodY controls the repressed expression of the *nuc* gene and extracellular proteases, both of which are considered important modulators of biofilm formation [117].

### Alternative sigma factor SigB

Sigma factors are important modulators of gene transcription, as they are responsible for guiding the RNA polymerase to specific promoters to initiate expression. *S. aureus* possesses four types of distinct sigma factors: σA, σB, σH, and σS [118]. σA is a housekeeping gene, as it is a sigma factor involved in regulating gene transcription. Alternative sigma factors are more associated with the regulation of genes with a functional role in response to stress and environmental changes. For instance, the role of σS is evident in the response to starvation or increased temperatures.

The alternative sigma factor σB (sigB) is another transcription factor with a significant role in responding to different environmental stresses [119].

The *rsbUVW-sigB* operon is the source of σB in *S. aureus*. RsbW and RsbV are both anti-σB proteins which downregulate the sigma factor and render it inactive when the surrounding conditions are favorable. On the other hand, RsbU is responsible for dephosphorylating RsbV and rendering it inactive, thereby delineating it as an indirect activator for σB. SarA can also indirectly regulate σB through the mazE promoter PmazE, which is part of a toxin-antitoxin system known as MazEF [120].

σB-mediated biofilm regulation extends from the initial stages of attachment to the final stages of dispersal. This sigma factor can regulate over 250 different genes, including the upregulation of adhesins such as FnbA and ClfA. It also downregulates genes involved in biofilm detachment, such as those responsible for the production of nucleases and proteases [121].

σB also regulates PIA-dependent biofilm formation. However, studies investigating this effect provide contradictory data. For instance, one study using MRSA mucosal isolate MA12 revealed that mutations in sigB led to decreased transcription of *ica* genes [43]. On the other hand, a study using CA-MRSA isolates, SH1000 and USA300, revealed that sigB-knockout did not affect ica-dependent PIA production. The bacterial inability to produce biofilm was rather associated with increased levels of Agr and subsequent protease enzymes [122]. This indicates that σB influence on PIA production can be considered strain-dependent.

### LytSR two-component system

The lytSR system is responsible for regulating cell death and lysis through utilization of certain bacteriophage-like holins. Holins are small proteins produced during bacteriophage infection of bacterial cells. They can induce cellular death by introducing pores in the cell membrane and disrupting the structural integrity of the bacterial cell wall [123]. In *S. aureus*, bacteriophage-like holins are produced from the *cidA* gene [124]. As previously mentioned, *cidA* is crucial for the exodus stage of the biofilm formation by controlling cell lysis and release of eDNA. The *lgrA* gene; part of the *lrgAB* operon, encodes for an anti-holin protein, which counteracts the effect of *cidA*. The LytSR two-component system is an important regulator of the cidA/lgr system. It upregulates the expression of *lgrAB* operon, and thus indirectly antagonizes *cidA*. The decrease of the membrane potential (ΔΨ) induces the expression of the LytSR system. Like most two-component systems (TCS), the LytSR system is composed of a sensor molecule, LytS, and a cognate response regulator, LytR [124]. These two components interact together to activate the *lrgAB* operon as a response to alteration in Ψ. This leads to decreased levels of eDNA, and accordingly, decreases biofilm formation.

### The LuxS/AI-2 system

The LuxS/AI-2 is an interspecies QS system, where it synthesizes and recognizes certain autoinducers in several bacterial species. The autoinducer for this system is a furanosyl borate diester molecule, released extracellularly to communicate with receptors on the surface of surrounding bacteria. AI-2 is synthesized through the conversion of s-adenosylmethionine into s-adenosylhomocysteine through the action of multiple methyltransferases. A specific nucleosidase called Pfs then cleaves the adenine molecule from the SAH to convert it into S-ribosylho- mocysteine. The LuxS acts as an AI-2 synthase, where it acts on the resultant product from Pfs, mainly SRH, and catalyzes its conversion into AI-2 [125].

The LuxS/AI-2 system is conserved in most Gram-negative and Gram-positive bacteria, including *S. aureus*. However, its influence on biofilm formation is still under investigation. One study showed that LuxS/AI-2 inhibits biofilm formation, acting as a negative regulator, and that AI-2 induced transcription of icaR, hence decreasing its levels [126]. Another study demonstrated significant increase in PIA production upon inactivation of the *luxS* gene, and that LuxS-negative strains exhibited higher levels of transcription of the biofilm positive regulator Rbf [127].

A study by Yu and colleagues demonstrated that the influence of the LuxS/AI-2 system on biofilm is concentration-dependent. They used an AI-2 precursor known as 4,5-dihydroxy-2,3-pentanedione on *S. aureus* biofilm, and discovered that the addition of the DPD molecule in nanomolar concentrations was responsible for changes in the biofilm via an icaR-dependent mechanism. Nevertheless, this effect disappeared upon increasing the concentration [126].

## Exploring the potential of natural and synthetic compounds as biofilm inhibitors for *S. aureus*: promising strategies for managing and treating biofilms

Understanding the role of natural and synthetic compounds in inhibiting biofilms created by *S. aureus* is important for devising effective strategies to control and treat biofilm-related issues. Numerous studies have investigated the potential of different compounds to target and disrupt *S. aureus* biofilms. This part of the review presents some key findings regarding the role of natural and synthetic compounds as biofilm inhibitors.

Natural compounds derived from various sources have demonstrated promising anti-biofilm activity against *S. aureus*. Plant-derived compounds, such as phenolic compounds like curcumin and resveratrol [128], essential oils including tea tree oil and cinnamon oil [129], and plant extracts such as cranberry extract [130] and garlic extract [131], have exhibited inhibitory effects on *S. aureus* biofilms. The anti-biofilm properties of these products primarily include inhibition of the biofilm’s polymer matrix formation, disruption of ECM production, suppression of cell adhesion and attachment, and reducing the production of virulence factors. These actions ultimately impede the QS network and interfere with biofilm development [131].

Phloretin; a natural phenol present on apple tree leaves, possesses a potent antimicrobial and antibiofilm activity against Gram-positive bacteria. In this regard, Lopes et al. found that phloretin had an inhibitory effect on biofilm formation in *S. aureus* RN4220 and SA1199B strains, achieving an inhibitory efficiency of up to 70% at low concentrations (1–256 μg/ml) [132]. Notably, the impact of phloretin on biofilm production was found to be dose-dependent [133]. Specifically, a concentration of 0.5 × MIC led to biofilm inhibition in 5 out of 8 strains, while an increase in biofilm production was observed when phloretin was used at 0.125 × MIC across all tested strains. Phloretin is suggested to inhibit biofilm via targeting efflux proteins. Baicalein is a flavone compound that can be isolated from Scutellaria baicalensis roots, which is a famous herb belonging to the Traditional Chinese Medicines (TCMs), and is used with other herbs to treat a wide range of disorders [122]. Chen et al. reported that baicalein at concentrations of 32 μg/mL and 64 μg/mL effectively inhibited 3- and 7-day *S. aureus* biofilm formation [134]. Furthermore, the combination of vancomycin and baicalein enhanced biofilm destruction, whereas vancomycin alone did not. The action of baicalein is attributed to the downregulation of the quorum-sensing system regulators *agrA*, *RNAIII*, and *sarA*, as well as gene expression of *ica*. *Ginkgo biloba* is another plant that has been used in TCM for many years. *Ginkgo biloba* L. exocarp extract (GBEE) was reported to exhibit a minimum inhibitory concentration (MIC) of 4 μg/mL and a minimum bactericidal concentration (MBC) of 8 μg/mL against both *S. aureus* and MRSA [135]. Furthermore, GBEE demonstrated a dose-dependent inhibition of biofilm formation by *S. aureus* and MRSA at concentrations of 4–12 μg/mL. Interestingly, 6 hours of GREE treatment was associated with downregulation of the expression of biofilm-associated factors *icaA* and *sarA*, whereas *sigB* was downregulated after 12 hours. Additionally, *icaR* was upregulated at 12 hours. Other natural compounds that demonstrate an inhibitory effect on *S. aureus* biofilms include erianin which inhibits the Sortase A transpeptidase and interferes with cell adhesion [136]. Moreover, wheat-bran was found to downregulate AHL level and inhibit QS [137] and isovitexin was found to inhibit the SpA and reduce the biofilm formation [137].

In addition, the development of semisynthetic and synthetic small organic molecules has provided a promising approach to address antibiotic tolerance and disrupt *S. aureus* biofilms. This field has gained significant interest in recent decades, as multiple semisynthetic and synthetic compounds were designed to inhibit *S. aureus* biofilms and interfere with crucial molecular targets. For instance, indolenine-substituted pyrazole derivative prevented biofilm formation and eliminated mature biofilms of MSSA and MRSA, indicating its potential as a candidate for further development as a biofilm inhibitor targeting *S. aureus* [138]. It had a minimum biofilm inhibitory concentration of 1.56 μg/mL and a minimum biofilm eradication concentration value of 6.25 μg/mL. Another synthetic benzimidazole molecule, known as antibiofilm compound 1 (ABC-1), has been identified through a small-molecule screening process [139]. ABC-1 has demonstrated the ability to prevent the formation of bacterial biofilms caused by *S. aureus* without impacting bacterial growth. ABC-1 treatment suppressed the expression of SpA, resulting in reduced biofilm formation. Moreover, ABC-1 also hindered the accumulation of PIA and eDNA on the cell surface. Other synthetic compounds which exhibited antibiofilm activity on *S. aureus* include 2-Phenylhydrazineylidene derivatives [140], which inhibit Sortase A-mediated bacterial adhesion, and halogenated phenazines that eradicate MRSA biofilms and quiescent persister cells [15].

## Nano-therapeutics as a magic bullet for biofilm eradication

Biofilm formation poses an increasingly significant threat as the gap widens between antibiotic resistance and the development of new antibiotics. To effectively combat biofilm formation, it is crucial to target both penetration and antibiotic resistance through a concurrently coupled targeted therapy. The concept of the magic bullet, introduced by Paul Erlich, emphasizes that drug targeting should deliver the drug to the right location, with the right concentration, and for the right duration [141] Broadly hypothesized, nanotechnology serves as the magic bullet for antibiofilm treatment [142].

The use of nanotechnology has emerged as a promising alternative strategy for treating bacterial and biofilm-mediated infections [143, 144]. Nanotechnology offers several advantages compared to traditional treatments. For instance, materials with greater surface-area-to-volume ratios exhibit improved reactivity without susceptibility to enzymatic degradation, drug toxicity, or untargeted delivery [145].

### The merits of nanoparticles (NPs) in fighting bacterial infections

**Fig. 5:**
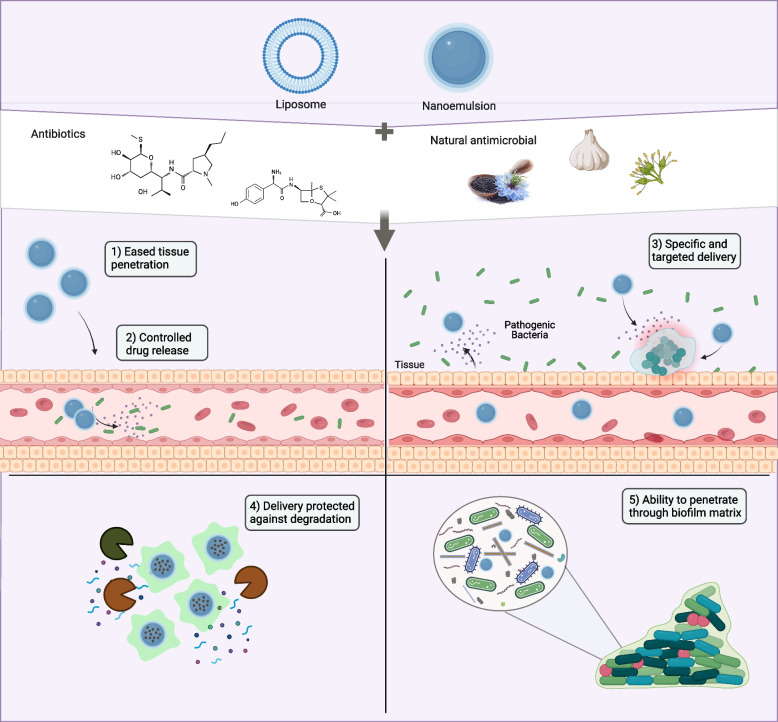
The use of nanoparticles such as liposomes and nanoemulsions as antibacterial-delivery systems

The size of a nanoparticle is in the range of 1–1000 nm [123–125]. Antibiofilm nano-therapy involves either the use of nanoparticles as delivery systems or the use of nanoparticles as the antibiofilm molecules themselves. Nanoparticles are either inorganic or organic particles. NPs can employ multiple bactericidal mechanisms (Fig. 5), including direct cell wall and/or cell membrane damage, generation of reactive oxygen species (ROS), and/or binding to intracellular components [146]. Furthermore, NPs can evade antimicrobial resistance (AMR) mechanisms and are less prone to resistance than conventional antibiotics [146]. Being nanosized, NPs penetrate tissues, facilitate easy drug uptake by cells, and efficiently deliver the drug. The uptake of nanostructures by cells is much higher than that of large particles with sizes ranging between 1 and 10 μm [147]. Furthermore, NPs allow sustained and controlled drug release with the desired intracellular therapeutic level of drugs and reduced or negligible side effects [143, 147].

Enrofloxacin-loaded docosanoic acid solid lipid nanoparticles increased the intracellular accumulation of enrofloxacin up to ~ 40-fold, and enhanced *Salmonella* killing inside macrophages. In another approach, colistin; a poorly permeable antibiotic, was formulated into liposomes functionalized with a bacterial-derived protein to promote internalization into eukaryotic cells and enhance oral bioavailability [146].

Nanoparticles can also encapsulate nutraceuticals, which include antioxidants, prebiotics, probiotics, herbal products, spices, polyunsaturated fatty acids, and many other compounds of natural origin [148, 149].

## Nano-technological strategies in combatting MRSA biofilm: breaking new grounds in antimicrobial advancements

### Metal-based nanoparticles

Inorganic metal-based nanoparticles have gained significant attention due to their antimicrobial action. These nanoparticles are renowned for their broad-spectrum antibacterial activity and intrinsic antibiofilm effects, presenting a promising treatment option for multidrug-resistant pathogens [150], [151]. Metal-based nanoparticles employ various mechanisms to eradicate bacterial biofilms and induce bacterial killing. For example, cationic nanoparticles interact with the EPS of the biofilm through surface non-covalent interactions [151]. Other metallic nanoparticles inhibit bacterial adhesion and halt biofilm formation at an earlier stage [152]. In addition to the passive impacts of metallic nanoparticles on biofilms, external stimuli such as photothermal therapy, photodynamic therapy, and magnetic fields have been utilized to synergize with metal nanoparticles in biofilm degradation [152, 153]. Once penetrating the biofilm matrix, metallic nanoparticles exert antibacterial effects by mechanically disrupting cell membranes, generating ROS, and interfering with cellular structures [144, 154]. Despite their promising activity, the widespread use of metallic nanoparticles faces multiple challenges. Recent studies have raised concerns about their safety, as metallic nanoparticles tend to accumulate in the biological system with a relatively low elimination rate [155]. This accumulation might be associated with necrosis, apoptosis, cytotoxicity, and multiple organ damage [156–158]. Furthermore, metallic nanoparticles, particularly the negatively charged ones, are linked to hemolysis and platelet aggregation, increasing the risk of organ damage [159]. Several studies have revealed that some metallic nanoparticles might cross the cytosol of body cells and interact alarmingly with metabolic enzymes, interfering with their functions [160]. Although different targeting and conjugation techniques enhance the selectivity of nanoparticles, the short and long-term toxicities of metal nanoparticles require extensive investigation. Additionally, the elevated cost of using high-throughput nanotechnology platforms and the variability and unpredictability of their physical properties are among the other limitations of large-scale production of metal-based nanoparticles [158, 161].

Silver nanoparticles (AgNPs) have garnered extensive attention recently due to their broad-spectrum antimicrobial effects and enhanced antibiofilm properties [162]. Ansari et al. discovered that low concentrations of AgNPs-coated surfaces significantly disrupted biofilm matrices in clinical MRSA isolates [163]. In another study, colloidal quasi-spherical AgNPs exhibited remarkable biofilm eradication (97% ± 1%) in MRSA isolates [164]. When tested in an infection model of *Caenorhabditis elegans*, quasi-spherical AgNPs demonstrated a substantial in vivo antibiofilm activity. Moreover, these nanoparticles were non-toxic and stable in suspension form, holding potential as a promising pharmacotherapeutic option for resistant MRSA [164]. Hamida and colleagues studied the effect of biogenic silver nanoparticles produced by Desertifilum sp. on clinical MRSA [165]. Biogenic silver nanoparticles were found to induce intense oxidative stress, leading to the oxidation of bacterial biomolecules and inhibition of biofilm formation. Unfortunately, while some studies indicated that AgNPs had no cross-resistance with clinically used antibiotics, prolonged exposure to these nanoparticles might lead to silver resistance and diminished efficacy through the acquisition of silver-resistance genes [166].

Gold-based nanoparticles (AuNPs) can also be used as antimicrobials; however, when used alone, they produce insignificant antibacterial and antibiofilm effects. Thus, AuNPs are used in conjunction with other active compounds or antibiotics for marked anti-biofilm activity against multiple resistant pathogens [167]. Hu et al. reported that pH-responsive gold nanoparticles were able to aggregate in the acidic microenvironment of MRSA biofilm [168]. Subsequently, acidic pH (around 5.5) changed the biofilm surface charge into a positive one, allowing efficient adherence of AuNPs to the negatively charged surfaces of the MRSA biofilm. Furthermore, AuNPs positively impacted photothermal biofilm eradication as they were able to absorb near-infrared (NIR) light irradiation and convert it into localized heat, leading to the thermal destruction of the MRSA biofilm. Since these nanoparticles were well-dispersed in slightly basic tissues, no damage to the healthy tissues surrounding the biofilm was observed. Likewise, protease-conjugated gold nanorods were able to inhibit exotoxin production and biofilm formation in *S. aureus* when subjected to NIR illumination, taking advantage of both hyperthermia produced by gold nanorods and the protease enzyme function [169]. Another study reported the design of AuNP-based multivalent aminosaccharides, with structural similarity to cell wall peptidoglycan, resulting in cell wall disruption and bacterial death [170]. Yang et al. reported that aminosaccharide-based gold nanoparticles exhibited outstanding in vitro and in vivo efficacy and biocompatibility when tested against MRSA-infected skin wounds [171].

Copper-based nanoparticles (CuNPs), such as copper and copper oxide NPs, exhibit effective anti-MRSA properties. However, high concentrations are needed to achieve optimum bacterial killing, which might be associated with toxicities in mammalian cells [172, 173]. Like silver NPs, CuNPs release Cu2+ ions, which disrupt cell membranes and interfere with cellular enzymes [174]. Kannan and colleagues formulated multilamellar liposomes encapsulating lipopeptide and CuNPs, which can efficiently diminish MRSA cells in both planktonic and biofilm states [175]. It was shown that liposomes enhanced the pharmacodynamics and pharmacokinetics of the formulation. Meanwhile, the observed synergistic effect of CuNPs and lipopeptide led to a 47% inhibition of EPS production in MRSA as well as a 75% increase in intracellular ROS generation. Marzban et al. managed to greenly synthesize CuNPs using water-soluble polysaccharides (SPs-CuNPs) [176]. These NPs exhibited MIC value of 250 μg/ml against MRSA. Also, they inhibited MRSA biofilms at 100 g/ml.

Other metal-based NPs include zinc oxide nanoparticles (ZnO NPs), which can damage proteins and lipids in bacterial cell membranes, leading to the leakage of cytoplasmic matter and bacterial death. They can also increase oxidative stress as a result of hydrogen peroxide formation [177]. They emit zinc ions, which inhibit the DapE protein involved in peptidoglycan synthesis, hence, ZnO suppresses biofilm formation at an initial stage [155]. In vitro analysis of ZnO NPs’ effect on MRSA and MSSA indicated that they were less effective than silver and copper nanoparticles [174–178]. In a recent study, the activity of ZnO NPs was tested on a collection of MRSA, VRSA, and linezolid resistant *S. aureus* [179]. The authors found that ZnO NPs had MIC values ranging from 128 to 2048 μg/ml, and the NPs concentration of 1024 μg/ml achieved 76.47% biofilm inhibition.

Titanium dioxide nanosystems (TiO2) eliminate biofilm formation mainly by generating ROS and oxidizing cellular lipids and proteins [180]. Recently, TiO2 has gained exceptional interest due to its stability and safety. Furthermore, it was recently revealed that TiO2 nanofibers can be used to coat different objects, such as medical devices and the surfaces of medical equipment, to prevent biofilm formation by nosocomial MRSA [181].

### Organic nanoparticles

Unlike metal-based NPs, organic NPs are prepared from biopolymers, such as proteins and lipids, or from synthetic organic molecules, such as poly (lactic-co-glycolic acid) (PLGA) and polycaprolactone (PCL) [182]. One key advantage of organic NPs over their inorganic counterparts is that they are biocompatible and biodegradable; therefore, they pose no risk of toxicity or provoking an immune response in the human body [183]. Additionally, organic NPs are also easy to prepare and are stable [183].

To fight MRSA, multiple organic NPs were prepared, loaded, and tested for the inhibition and eradication of MRSA biofilm. Some organic NPs have intrinsic antibacterial properties due to their cationic properties, which help them bind to bacterial cell membranes and cause leakage of cellular components that result in bacterial cell death [184]. Chitosan, for example, may prevent adhesion of MRSA to surfaces due to its effect on the hydrophobicity of bacterial cell membranes, thus inhibiting biofilm formation by around 30% [185]. It can also be loaded with different agents, such as antibiotics and inorganic metals, for synergistic effects. For example, the photosensitizer methylene blue showed better eradication of MRSA biofilm via photodynamic inactivation when combined with low-molecular-weight chitosan NPs [164]. Quaternized chitosan loaded on titania nanotubes showed promising results for biofilm inhibition in MRSA [186], and demonstrated significant biofilm inhibition activity against MRSA biofilms [187]. While antimicrobial peptides (AMP) can be toxic, expensive, and of low stability, an ultrashort AMP (RBRBR) was successfully loaded on a chitosan-based nanosystem and proved its efficacy, safety, and enhanced selectivity to bacterial cells, with antibiofilm activity [188]. Another AMP is the synthetic antimicrobial octapeptide (IKFQFHFD) that was formulated as a pH-switchable hydrogel with nanofiber networks, and both photothermal cypate and collagen promoter proline were loaded into the hydrogel system. The biocompatible supramolecules totally eradicated MRSA biofilm and promoted wound healing [189].

Although nitric oxide (NO) is an effective antibiofilm with wound healing properties, the pharmaceutical formulation of NO is challenging due to its gaseous nature and short half-life. Hasan et al. succeeded in loading NO into PEI/NONOates-doped PLGA nanoparticles and tested the system in vivo with total eradication of MRSA biofilm and less bacterial burden [190]. Biguanide-based polymetformin, together with tannic acid and Pluronic F-127, are biocompatible NPs that were designed not only to eradicate MRSA biofilm, but also to have bactericidal effect on live sessile bacteria to prevent colonization [191]. Combination therapy, in terms of enzyme modification and NIR, were applied to both eliminate MRSA biofilm and accompanying inflammation [192].

### Combination antimicrobials loaded in NPs

Combination therapy serves as a promising and easy-to-implement alternative option. Multiple studies have investigated the applicability and therapeutic efficacy of using combinations of different antibiotics or combining antibiotics with other agents like phages, antimicrobial peptides, and nanoparticles [193]. Since resistance rates are higher in bacterial cells within biofilms compared to their planktonic counterparts, it is critical to examine the biofilm inhibition properties of these combinations [194]. Although the use of two or more antibiotics is currently employed in the treatment of biofilm-mediated infections, the associated toxicity and AMR to these combined antibiotics urge the search for other combinations [195]. Owing to their properties, NPs are ideal carriers for the delivery of antibiotics and other active molecules, which can conquer persistent biofilms and prevent bacterial colonization [144].

Combinations of nano-encapsulated oregano oil with both ciprofloxacin and gentamicin were evaluated against MRSA skin infections, and it was reported that both combinations significantly inhibited biofilm development compared to the effects produced by each antibiotic alone [196]. Regarding their antibacterial effects, the gentamicin-containing combination exhibited synergistic anti-staphylococcal activity, whereas a ciprofloxacin-containing combination had an additive effect. Similarly, soyaethyl morpholinium ethosulfate cationic nanostructured lipid carriers were loaded with oxacillin and tested against MRSA [197]. This combination exhibited a synergistic antibiofilm effect, decreasing MRSA biofilm thickness from 31 to 13 μm compared to oxacillin (25 μm) or nanocarriers (18 μm) alone. Xiao et al. synthesized dual-stimuli vancomycin-encapsulated nanoparticles to target vancomycin-intermediate *S. aureus* [198], consisting of zeolitic imidazolate frameworks-8 with polydopamine on the surface. This combination responded to both pH and photothermal activation using NIR light illumination to control the delivery of vancomycin and synergistically destroy both planktonic and biofilm bacteria. Vancomycin loaded on the dual organic nanosystem, mPEG-b-PCL and G1-PEA, showed much higher efficiency in MRSA biofilm destruction compared to vancomycin alone.

### Quorum inhibiting nanomaterials

Anti-virulence therapy is an approach that targets pathways contributing to pathogenesis. In *S. aureus*, QS modulates the expression of staphylococcal enterotoxin C, delta-toxin, and Panton-Valentine, all of which contribute to the virulence of the bacteria [199]. As previously discussed, QS is a complex signaling cascade involving several proteins and nucleic acids that collaboratively regulate the transcription of virulence genes. Targeting a signaling cascade is a promising treatment approach, where each component can be regarded as a potential candidate [200].

Quorum quenchers are molecules targeting the QS signal, leading to its inactivation. The mode of inactivation varies, as well as the type of quorum quencher (either enzymes or compounds) and the target. They can act through signal cleavage or competitive inhibition [201]. The quorum inhibitors, however, interfere with the quorum signaling pathways leading to their disruption. Therefore, this interference can be at different stages of the signaling pathway. For instance, quorum inhibitors interfere with the synthesis of the autoinducers, the exchange of the autoinducers between cells, and the process of perceiving and transducing the signal that occurs through interactions with transcriptional factors [201]. For example, savirin (*S. aureus* virulence inhibitor) is a small molecule inhibitor with the ability to block the binding of the AgrA protein to the respective promoter sites, thus preventing further stimulation of the P2, and consequently decreasing the expression of the controlled genes [202]. Another inhibitor is an antisense oligonucleotide that targets the agrA gene and one that targets the RNAIII molecule. These oligonucleotides are known as locked nucleic acids (LNA) synthesized by modifying ribonucleotides with an additional bridge between a carbon and an oxygen molecule. LNAs are conjugated with cell-penetrating peptides to facilitate access to their targets [203].

Quorum inhibitors can be naturally extracted from plants and fungi. For example, ambuic acid is a fungal metabolite that was found to inhibit quorum signaling in a dose-dependent manner. Intradermal administration of 25 μg of ambuic acid prevented formation of ulcers in mice with MRSA skin infections [204]. Ambuic acid targets the agrB and decreases expression of the RNAIII molecule. Consequently, it inhibits AIP synthesis and production of alpha-toxin. Another example is baicalein. Baicalein is a primary flavonoid used in TCM, discovered in the roots of Scutellaria baicalensis It is used as a quorum sensing inhibitor. It inhibits AgrD signaling molecule when it is combined with AgrC. It is suggested that baicalein blocks the RNAIII activating peptide, therefore, it can suppress the phosphorylation of the downstream cascade, and inhibit the expression of the genes responsible for biofilm formation [134].

Nanomaterials are loaded with quorum-sensing inhibitors (QSIs) to allow them to penetrate to the inner layers of the biofilm. A study in 2019 used graphitic hollow carbon nitride nanospheres as loading systems and compared the effect of dual cargos; QSIs and antibiotics. These nanospheres were loaded with luteolin (a natural falvone extracted from plants) and ampicillin. The nanospheres were capped with hyaluronic acid, which is later decomposed by Hyal inside the bacterium to allow the nanospheres to release its cargo. Treatment attempted a multistage release to sensitize the bacteria to the therapeutic effect of ampicillin [205]. The effect of the antibiotic was complemented with the QSIs, which were supported by photodynamic therapy to induce production of ROS and enhance the QSIs antibiofilm effect. These nanospheres were tested on mice models infection with *S. aureus* [206].

In addition to delivery, nanomaterials can act as quorum quencher intrinsically without the need for loading. For instance, silver nanoparticles synthesized from Cymbopogan citratus leaf extract were able to inhibit QS in *S. aureus* biofilms [207]. Another study used pegylated silver coated carbon nanotubes and showed that treatment of *S. aureus* biofilms with these nanotubes resulted in a decrease in the expression of *sdiA* gene (a quorum sensing gene), and other subsequently activated virulence genes such as *safC*, *sseG*, *sseA*, and *ychP *[208].

**Fig. 6:**
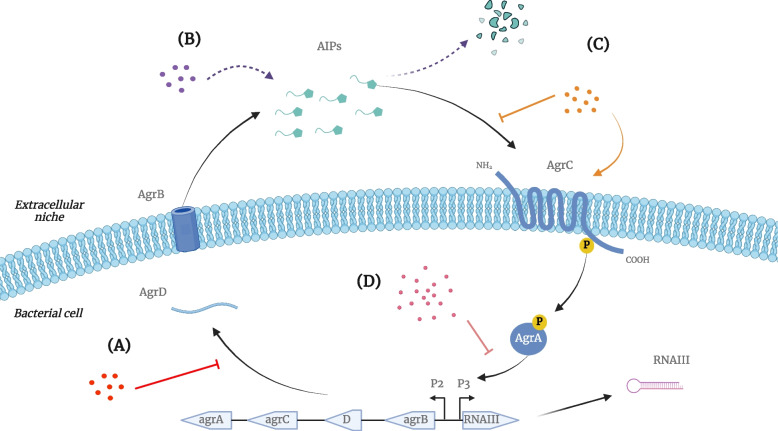
Mechanism of action of quorum-sensing inhibitors

QSIs have a broad action spectrum illustrated in Fig. 6. QSIs can interfere with the synthesis of the AIP (e.g., Ambuic acid), or act as degradative enzymes and destroy produced AIPs (e.g., lactonases). Additionally, QSIs can act as competitive inhibitors and block binding of the AIPs to their respective receptors (e.g., Cochinmicin), or they can prevent the dimerization of the receptor altogether (e.g., 3-Tetradecanoyltetronic acid). QSIs can prevent the activation of AgrA and prevent its binding to the DNA site (e.g., 2-(4-Methyl-phenyl)-1,3- thiazole-4-carboxylic acid) [200].

Despite the vast diversity of QSIs and their mechanisms of action, studies applying nanotechnology for the targeted quorum quenching therapy on MRSA biofilms are limited. Therefore, exploring novel nano-therapeutics together with targeted quorum quenching therapies against MRSA biofilms presents an innovative approach, paving the way for enhanced treatment efficacy and development of promising strategies to combat biofilm-mediated resistance.

## Conclusion and future prospective

Combating biofilm is a crucial strategy in preventing the dissemination of antimicrobial resistance. The presence of two additional stages, multiplication and exodus, in MRSA biofilm formation reveals opportunities for targeted interventions at various stages of the process. Quorum sensing emerges as an intriguing mechanism to inhibit and effectively eliminate biofilms. While disrupting biofilm architecture is significant, eliminating sessile bacterial cells is essential to prevent colonization on other surfaces or within body organs. In this regard, nanoparticles exhibit distinct physicochemical characteristics that enable the delivery of antimicrobial and antibiofilm agents throughout the complex biofilm architecture.

Although metal-based nanoparticles have shown effectiveness, their non-degradability and limited elimination by the human body pose challenges related to accumulation and toxicity. Organic nanoparticles, in conjunction with quorum quenchers, can be considered a promising approach against biofilms, representing a potential magic bullet. With nanoparticles already established in therapy, the anticipation is for more FDA-approved antibiofilm nanotherapeutics to enter the market, addressing challenges associated with prosthetics, implants, and wound infections.

In addition, future research for next-generation MRSA antibiofilm strategies should explore advanced molecular-targeted therapies focusing on specific molecular mechanisms involved in MRSA biofilm formation. For instance, identifying novel compounds and developing innovative quorum inhibitors with the potential to interfere with the quorum sensing system, possibly targeting autoinducing peptides or their receptors, are promising avenues for exploration. Developing biocompatible nanoparticles with enhanced degradability within the human body is also crucial as a cornerstone of future research on antibiofilm therapies. This paves the way for safer and more sustainable nanotherapeutics to combat MRSA biofilm. Furthermore, exploring the synergistic effects of combining different antibiofilm agents, including nanoparticles and quorum inhibitors, could enhance the overall efficacy of biofilm eradication. These targeted therapies could provide innovative avenues for anti-virulence and antibiofilm strategies to combat MRSA-mediated infections, mitigate the risk of resistance development, and improve treatment outcomes.

## Data Availability

No datasets were generated or analysed during the current study.

## Abbreviations

AIP: Auto-inducing peptide
agr: Accessory gene regulator
AMP: Antimicrobial peptide
AMR: Antimicrobial resistance
CA-MRSA: Community-acquired methicillin-resistant *Staphylococcus aureus*
AgNPs: Silver nanoparticles
AuNPs: Gold-based nanoparticles
CNA: Collagen-binding adhesin
CuNPs: Copper-based nanoparticle
CWA: Cell wall-anchored protein
ECM: extracellular matrix
EPS: Extracellular polymeric substances
HGT: Horizontal gene transfer
LA-MRSA: Livestock-associated methicillin-resistant *Staphylococcus aureus*
LNA: Locked nucleic acid
MFS: major facilitator superfamily
MIC: Minimum inhibitory concentration
MSCRAMMs: Microbial surface components recognizing adhesive matrix molecules
HA-MRSA: Healthcare-associated methicillin-resistant *Staphylococcus aureus*
NIR: Near-infrared
NPs: Nanoparticles
PBP: Penicillin-binding proteins
PCL: Polycaprolactone
PIA: polysaccharide intracellular adhesin
PLGA: Poly (lactic-co-glycolic acid)
PSM: Phenol soluble modulin
QS: Quorum sensing
QSI: Quorum-sensing inhibitors
ROS: Reactive oxygen species
*S. epidermidis*: *Staphylococcus epidermidis*
TCM: Traditional chinese medicine
TSST-1: Toxic shock syndrome toxin-1
VRSA: Vancomycin-resistant *Staphylococcus aureus*
ZnO NPs: zinc oxide nanoparticles
